# Green Synthesis of Selenium and Tellurium Nanoparticles: Current Trends, Biological Properties and Biomedical Applications

**DOI:** 10.3390/ijms22030989

**Published:** 2021-01-20

**Authors:** Marjorie C. Zambonino, Ernesto Mateo Quizhpe, Francisco E. Jaramillo, Ashiqur Rahman, Nelson Santiago Vispo, Clayton Jeffryes, Si Amar Dahoumane

**Affiliations:** 1 School of Biological Sciences and Engineering, Yachay Tech University, Hacienda San José s/n, San Miguel de Urcuquí 100119, Ecuador; marjorie.zambonino@yachaytech.edu.ec (M.C.Z.); ernesto.quizhpe@yachaytech.edu.ec (E.M.Q.); francisco.jaramillo@yachaytech.edu.ec (F.E.J.); nvispo@yachaytech.edu.ec (N.S.V.); 2 Center for Midstream Management and Science, Lamar University, Beaumont, TX 77710, USA; arahman2@lamar.edu; 3 Center for Advances in Water and Air Quality & The Dan F. Smith Department of Chemical Engineering, Lamar University, Beaumont, TX 77710, USA; cjeffryes@lamar.edu; 4 Department of Chemical Engineering, Polytechnique Montréal, C.P. 6079, Succ. Centre-ville, Montréal, QC H3C 3A7, Canada

**Keywords:** SeNPs, TeNPs, nanofactories, biosynthesis, biomass, mechanistic aspects, bioactivity, bioapplications, sustainability

## Abstract

The synthesis and assembly of nanoparticles using green technology has been an excellent option in nanotechnology because they are easy to implement, cost-efficient, eco-friendly, risk-free, and amenable to scaling up. They also do not require sophisticated equipment nor well-trained professionals. Bionanotechnology involves various biological systems as suitable nanofactories, including biomolecules, bacteria, fungi, yeasts, and plants. Biologically inspired nanomaterial fabrication approaches have shown great potential to interconnect microbial or plant extract biotechnology and nanotechnology. The present article extensively reviews the eco-friendly production of metalloid nanoparticles, namely made of selenium (SeNPs) and tellurium (TeNPs), using various microorganisms, such as bacteria and fungi, and plants’ extracts. It also discusses the methodologies followed by materials scientists and highlights the impact of the experimental sets on the outcomes and shed light on the underlying mechanisms. Moreover, it features the unique properties displayed by these biogenic nanoparticles for a large range of emerging applications in medicine, agriculture, bioengineering, and bioremediation.

## 1. Introduction

Nanotechnology has become one of the most promising interdisciplinary technologies, connecting physics, chemistry, biology, materials science, electronics, and medicine [1]. The quantity of engineered nanoparticles (NPs) is expected to increase significantly in the years to come as they receive growing global attention due to their attractive properties, multifunctionalities, unique characteristics, and innovative applications in different industrial and scientific domains [2,3,4,5,6]. Several physical and chemical methods have been extensively explored to fabricate NPs, such as laser ablation [7,8], coprecipitation [9,10], hydrothermal route [11,12], solvothermal route [13,14], sol-gel process [15,16], polyol process [17,18], electrochemical methods [19,20], sonochemistry [21,22], and microwave-assisted methods [23,24]. However, the use of toxic chemicals and/or the generation of harmful byproducts limit their application in clinical fields. Thus, materials scientists rely on a plethora of precursors and reducing/stabilizing agents from biological resources to produce environmentally friendly NPs to lower or eliminate the use and generation of hazardous chemicals [25,26,27]. Such biosystems include natural biomolecules [28,29,30,31], plants [32,33,34], algae [35,36,37,38,39,40], bacteria [41,42], yeast and fungi [43,44]; these biological entities exhibit high reductive capacities due to the presence of enzymes, proteins, lipids, sugars, and metabolites. Overall, the biological-mediated synthesis of metallic and metalloid nanoparticles is a single-step, bioreductive process that follows a bottom-up approach and involves the reduction of metal ions dissolved usually in aqueous solutions at room or mild temperature and atmospheric pressure [33,45,46].

Nanoparticles have remarkable advantages over bulk materials, such as a larger surface area, higher surface energy, spatial confinement and reduced imperfections [47]. Their features, such as the size, morphology, chemical composition, surface functionality, and crystallinity, play an important role in determining their potential applications in numerous fields, such as biomedicine, nanobiotechnology, agriculture, pharmacology, optoelectronics, etc. [48,49,50]. Over the past few years, selenium and tellurium have become chalcogenides of great interest owing to their unique photoconductive and thermoconductive properties [51]. They are known as “E-tech” elements with characteristics similar to that of sulfur and are fundamental constituents of photovoltaic solar panels, electronic devices, and alloys [51,52].

Selenium is an essential trace element for life [53,54]. It is an allotropic nonmetal usually red and grey present in nature under three forms: amorphous, crystalline trigonal with helical chains, and crystalline monoclinic (α, β, γ) with Se8 rings [6]. The synthesis of selenium nanoparticles (SeNPs) by microorganisms and plants induces variations in their crystallinity, morphology, and size due to the diversity of the followed biological methodologies, reducing enzymes and biosurfactants [55]. Although some investigations have reported the biosynthesis of SeNPs under aerobic and anaerobic conditions, aerobic microorganisms have generated the ideal outcomes [56]. The process typically reduces selenite (Se(IV)) or selenate (Se(VI)) species into elemental selenium (Se(0)). Se-based nanomaterials exhibit chemotherapeutic and chemopreventive features, antioxidant properties, low cytotoxicity, and anticancer efficacy, making them a useful tool in nanomedicine [57,58]. They also have a strong, dose-dependent antimicrobial effect on various microorganisms’ growth and propagation [56].

**Figure 1:**
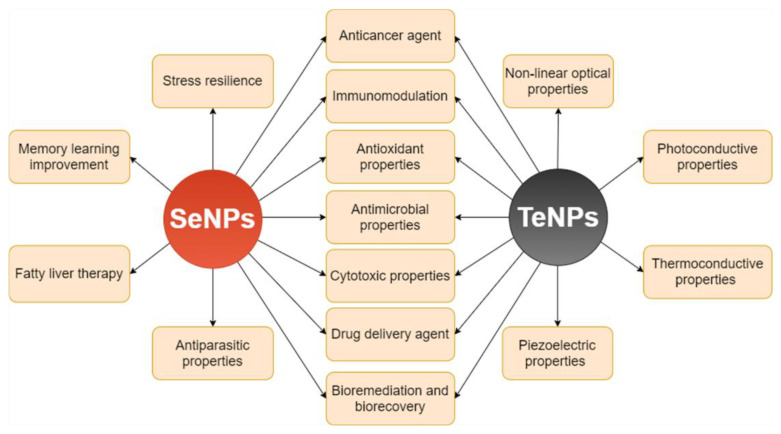
Applications of selenium nanoparticles (SeNPs) and tellurium nanoparticles (TeNPs).

Tellurium is a metalloid present in nature as a soluble oxyanion under four oxidation states: −2 (H_2_Te), +2 (TeO_2_^2−^), +4 (TeO_3_^2−^), and +6 (TeO_4_^2−^). It can be toxic in very low concentrations (1 μg mL^−1^) [59]. Recently, the conversion of tellurite to black elemental tellurium including extra/intracellular accumulation, volatilization, and methylation, has piqued the interest of researchers [60]. Tellurium nanoparticles (TeNPs) have become of interest in research and industry due to their excellent biocompatibility [61], antimicrobial, antioxidant and anticancer activity [62,63], and their ability to reduce cholesterol and triglyceride levels [64]. The high efficiency of microorganisms to transform metalloid oxyanions to less toxic elemental forms results in toxicity reduction and increased selenium and tellurium bioavailability [65]. Moreover, the same microorganisms provide exceptional bioremediation tools and technological applications due to their ability to biorecover the cations of these metalloids and promote the subsequent production of Se and Te nanomaterials [51,66,67,68,69,70]. The principal applications of biogenic SeNPs and TeNPs are summarized in Figure 1.

The present review aims at providing a comprehensive insight upon the emerging routes implemented for the biosynthesis of SeNPs and TeNPs using various microorganisms and plants via different methodologies. It also elaborates on the underlying mechanisms that govern these bioprocesses, describes the unique biological properties of these metalloids’ nanomaterials, and discusses their diverse applications in the biomedical field.

## 2. Green Synthesis of Inorganic Nanoparticles Using Microorganisms

The holy grail in nanotechnology consists in elaborating cost-effective and environmentally friendly approaches for the synthesis of nanomaterials that modulate their size, morphology, assembly, and colloidal stability [71]. The biosynthesis of inorganic nanoparticles is generally implemented in aqueous media at room temperature or mild heating and atmospheric pressure [26]. Those are simple conditions that engage the production of high-quality nanomaterials. In that sense, these NP biosynthetic methods that rely on microorganisms, such as bacteria, fungi, microalgae, yeast and viruses, and plants are fully eco-friendly approaches [42]. These microbial and plant-assisted methodologies provide easy, inexpensive, and nontoxic routes to yield NPs that exhibit a diversity of sizes, shapes, and composition along with unique physicochemical attributes and outstanding biological properties.

Nature has devised several reliable, cost-effective, nontoxic, clean, and ecofriendly biological techniques to produce SeNPs and TeNPs [72,73]. Green nanotechnology employs natural biological resources, such as bacteria, fungi, yeast, algae, plants, and viruses, and, most often, water as the solvent. To achieve the fabrication of monodispersed, highly stable NPs with a desired size and controlled morphology, the biomolecular machinery availability is needed [74]. The main benefit is that microorganisms are effective tools that act as nanofactories avoiding thus the use of and/or generation of harsh, toxic chemicals. They also have the ability to accumulate and detoxify heavy metals due to various reductase enzymes that reduce metal salts to metallic nanoparticles with a narrow size distribution and, therefore, less polydispersity [75,76]. Biological processes usually occur at mild conditions, i.e., ambient temperature and atmospheric pressure, and do not require skilled professionals nor sophisticated equipment making them amenable to controlled and scale-up procedures [74]. However, they also present some limitations related to NP composition, crystallinity, morphology, and size distribution.

Recently, the extra- and intra-cellular microbial production of metallic/metalloid NPs have been studied [27,33,41,43,44,45,77,78,79]. In extracellular formation, the added metal salts are transformed into NPs in the culture broth or attached to the cell membrane. Conversely, the intracellular process first transports the metal ions through the cell membrane, i.e., internalization, to the cell interior where the nanoparticles are formed. Then, these internally formed NPs are released to the supernatant using several procedures, such as the cell lysis, to be recovered and purified [72,74,80]. The following sections describe the outstanding role played by different microorganisms, namely bacteria, fungi and yeast, and plants in the biosynthesis of SeNPs and TeNPs.

## 3. Parameters Affecting the Green Synthesis of Metalloid Nanoparticles

Various factors, such as the precursor, biomass type, temperature, pH, and reaction time, govern the production and stabilization of SeNPs and TeNPs by microorganisms. The pH is an important factor that determines the shape, size, and composition of the NPs [80,81]. For instance, Wu et al. reported the formation, at pH 8, of effectively dispersed spherical SeNPs of 60 nm in diameter in epigallocatechin-3-gallate (EGCG). However, the protonation of the EGCG in acidic conditions (pH 1.0) rapidly induced the aggregations of these NPs as their dimensions reached 300 nm within the first 3 min resulting eventually in the loss of their nanoscale features [82]. According to Akçay and Avcı, the maximum yield occurred at pH 7 and 8 [83] while Kuroda et al. reported the optimum reduction rate at pH values of 6–9 for selenite and 7–9 for selenate [84]. Wadhwani et al. demonstrated the synthesis of SeNPs in a pH range of 4–10 [58]. No synthesis occurred at pH 2 and 1.5 mM of sodium selenite due to the presence of less functional groups that are required for the reduction process. The precursor concentration can also control the NP shape and size. For example, the same study by Wadhwani et al. proved that spherical and rod morphologies of the SeNPs appear at 3.0 mM Na_2_SeO_3_ while only spheres are observed at 1.5 mM of the same precursor [58].

Green approaches for the synthesis of SeNPs and TeNPs are cost- and energy-efficient, requiring lower temperatures compared to their chemical or physical counterparts [58]. The temperature is found to be a factor that leads to the formation and then aggregation of SeNPs [85]. For instance, the reduction process occurs at temperatures up to 40 °C using *Acinetobacter* sp. SW30 and higher temperatures (around 80 °C and 100 °C) may lead to the aggregation of the SeNPs into nanorods [58]. It is relevant to indicate that, in the case of bacteria, elevated temperatures (>45 °C) may block the normal biosynthesis of SeNPs [86]. Likewise, high temperatures (over 60 °C) and low temperatures (below 25 °C) reduce the efficiency of inorganic NP production using fungi [87,88]. Moreover, the incubation time plays a significant role in the quality and morphology of the NPs. In the case of most bacteria, the average incubation time ranges from 24 to 72 h, but long incubation periods may cause NPs to aggregate, grow, or shrink [89]. The properties of NPs may have a lifetime, but extended exposure times can induce metastable changes to the surface morphology, crystallinity, and optical absorption of nanostructures [90].

The concentration of precursors and reducing/surfactant agents are also critical to control the growth and morphology of the nanoparticles [26,91,92,93]. The precursor concentration can have a strong influence on the color intensity and rate of change during the NP formation process [94,95]. Se (Na_2_SeO_4_, Na_2_SeO_3_, SeO_2_) and Te (Na_2_TeO_3_, K_2_TeO_3_) precursors along with the pH and reaction time are tuned to produce metalloid nanostructures of different sizes [49,96] and shapes (e.g., SeNPs, Te nanorods (TeNRs), Te nanowires (TeNWs), and Te nanotubes (TeNTs)) [97,98]. Additionally, the size of SeNPs is determined by the initial precursor concentration [99]. The tolerance towards selenium oxyanions can be evaluated by exposing the microorganisms to different precursor concentrations. For example, Presentato et al. evaluated the bioconversion yield and rate of 0.5 and 2 mM of SeO_3_^2−^ into thermodynamically stable Se(0) nanostructures considering unconditioned and conditioned physiological states of the actinomycete *Rhodococcus aetherivorans* BCP1 [99]. The results showed that the initial precursor concentration had a strong effect on the size and size evolution of the obtained SeNPs. For instance, the smallest Se NPs that are obtained at the lowest concentration evolve to form Se nanorods (SeNRs). On the other hand, the longest SeNPs obtained at the highest concentration eventually form the shortest SeNRs. The strain *Phomopsis viticola* has the same degree of inhibition, in terms of biomass production, when incubated in the presence of SeO_3_^2−^ or TeO_3_^2−^ [100] whereas two strains of *Aspergillus*, *A. flavus* DSMZ 1959 and *A. parasiticus* DSMZ 1300, were less inhibited by SeO_3_^2^ compared to TeO_3_^2−^ [100]. However, Wang et al. found that different sodium selenite concentrations did not affect the size and morphology of the produced SeNPs using *Bacillus subtilis* [101].

To optimize SeNP bioproduction, the selenium precursor concentration (sodium selenite) varied from 10 to 30 mM and the impact of the pH and reaction time was assessed [102]. Besides, statistical optimization techniques might be used for the design of the experiment, such as the response surface methodology (RSM) [102,103]. Overall, the yield of NP synthesis has a direct correlation with the precursor concentration: the higher the concentration, the greater the production. Moreover, it can be suggested that the lower the precursor concentration and temperature, the smaller the size of produced NPs (*vide infra*).

## 4. Techniques of Characterization

The characterization of metalloid NPs is needed to correlate their physicochemical properties to their biological effects and toxicity [49,104,105,106,107]. The initial physicochemical characterization of these NPs is carried out by using a myriad of routine lab techniques to analyze their shape, size and size distribution, porosity, surface chemistry, crystallinity, and dispersion pattern [108]. The most widely used techniques include UV-visible (UV-Vis) spectroscopy, luminescence spectroscopy (LS), scanning electron microscopy–energy dispersive X-ray spectroscopy (SEM-EDX), transmission electron microscopy (TEM), Fourier transform infra-red spectroscopy (FT-IR), X-ray diffraction (XRD). XRD confirms the presence of NPs and determines their lattice structure, crystallinity, and crystallite size using the Debye–Scherrer equation [21]. Electron microscopy techniques, such as TEM and SEM, enable the study of NP shape and size to deduce their size distribution along with elemental composition (EDX) [21,109]. According to Kapur et al., magnified field emission scanning electron microscopy (FESEM) images provide information about the nature and composition of the NPs [108]. The FTIR is an efficient technique that provides reproducible analyses used to reveal the presence of functional groups at the NP surface. These groups may be involved in the reduction of the metal ions and/or the NP capping that ensures the colloidal stability [58,95]. In addition to determining the surface charge (z-potential) of the NPs, the dynamic light scattering (DLS) provides the NP hydrodynamic diameter and good insight into their stability/aggregation by measuring their Brownian motion [108]. The atomic force microscopy (AFM) provides quantitative information about length, width, height, morphology, and surface texture of NPs through a tridimensional visualization [56].

## 5. Microbial Biosynthesis of Selenium Nanoparticles

### 5.1. Using Bacteria

In recent years, the biosynthesis of Se-containing NPs using bacteria has been reported as a new environmentally friendly route that offers tremendous advantages, such as easy handling, short synthesis times, and simple genetic manipulation [101]. Various bacteria reduce inorganic selenite (SeO_3_^2−^) or selenate (SeO_4_^2−^) to elemental red selenium Se(0) nanoparticles of various morphologies including spherical, hexagonal, polygonal, and triangular ones [109]. The academic community has extensively explored the aerobic and anaerobic bacteria involved in the production of SeNPs (Table 1) through various reduction pathways under both aerobic and anaerobic conditions [56,73,110,111,112,113]. However, further investigations are required to fully determine the underlying biochemical pathways and the biochemicals that govern these processes.

The following species have been screened under aerobic conditions: *Streptomyces minutiscleroticus* M10A62 [131], *Comamonas testosteroni* S44 [143], *Lactobacillus* sp., *Bifidobacterium* sp. and *Streptococcus thermophilus* [146], *Enterobacter cloacae* Z0206 [140], *Azospirillum brasilense* [125] and the gram + bacteria *Bacillus* strains: *Bacillus* sp. MSh-1 [117,147], *B.* *subtilis* [101], and *B. cereus* [127]. On the other hand, several species of anaerobic bacteria have been screened for their ability to promote the production of SeNPs, such as *Shewanella* sp. HN-41 [116], *S. oneidensis* MR-1 [141], *Stenotrophomonas bentonitica* [71], *Alishewanella* sp. WH16-1 [75], *Vibrio natriegens* [133], and the facultative anaerobic bacteria *L. casei* 393 [134,148]. Moreover, anaerobic upflow sludge blanket reactors are used to fabricate SeNPs [53,149,150,151]. Besides, some species are able to biosynthesize SeNPs under aerobic and/or anaerobic conditions, such as *Azoarcus* sp. CIB [121].

The aerobic Se-reducing bacteria are simpler, faster, and more effective synthesizers of SeNPs as they grow rapidly and produce more cells [123]. They also possess greater advantages in agriculture and bioremediation over anaerobic bacteria since the soil and water treatment occurs aerobically [152,153,154]. Other benefits lie in their ability to identify the functional microbiota and the molecular homeostatic mechanisms responsible for Se oxyanion reduction. For example, in the case of the aerobic strain *C. testosteroni* S44, which can resist the toxicity of some heavy metal cations, such as Cu^2+^, Zn^2+^, As^4+^, and Se^4+^, the reduction of Se(VI) to SeNPs is carried out by the sulfite reductase (CysIJ) enzyme in the sulfate assimilation pathway [123]. This pathway has been suggested to be the general mechanism of selenate (Se(VI)) reduction in aerobic organisms related to the selenium biocycle. Moreover, the Cr(VI) reductase (known as CsrF) in the genome of *Alishewanella* sp. WH16-1 has been reported as a novel bacterial aerobic selenite reductase [75]. Due to its similarities with the structure and reduction activity of the flavoenzymes ChR, FerB and ArsH, CsrF may also act as a Se(IV) reductase.

In anaerobic bacteria, Se(VI)/Se(IV) reduction can occur on the cell surface via a two-step process; first, Se(VI) is reduced to Se(IV), then Se(IV) is reduced to subsequently give rise to SeNPs [155]. Conversely, in aerobic bacteria, it is more challenging to reduce Se-oxyanions on the surface of cells due to the tendency of oxygen to accept the electrons prior to Se(IV) [123,156]. Therefore, the reduction occurs intracellularly and then Se(0)/SeNPs are exported extracellularly by cell lysis [53,157], rapid expulsion pathway [158], efflux via a vesicular secretion system [155], vesicular transport [159], and hyphal lysis or fragmentation [160]. Nevertheless, the specific efflux system is still unknown.

**Figure 2:**
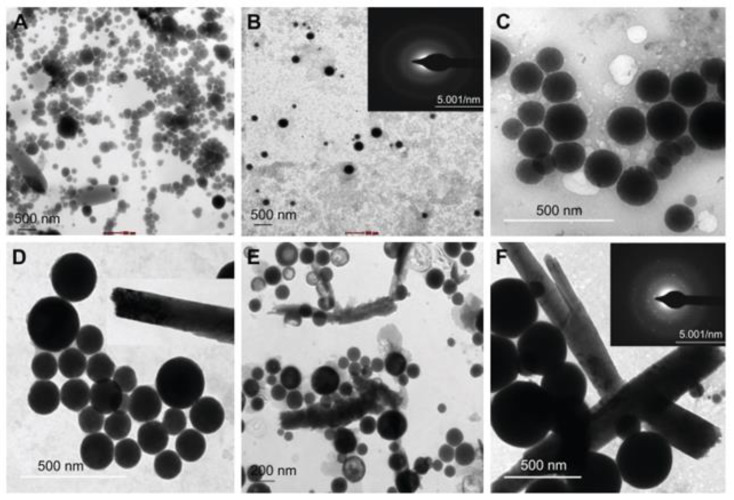
Transmission Electron Microscopy images of biogenic SeNPs synthesized by incubating the cell suspension of *Acinetobacter* sp. at 37 °C with 1.5 mM Na_2_SeO_3_ at: (**A**) pH 6, (**B**) pH 7 and (**C**) pH 9. TEM micrographs of the same experiment when the Na_2_SeO_3_ concentration is brought to 3.0 mM at (**D**) pH 6, (**E**) pH 7, and (**F**) pH 9. Reproduced from [58] with permission from Dove Medical Press.

Estevam et al. produced SeNPs using *Staphylococcus carnosus* TM300 that were harvested by first sonicating the pellet and then separating the NPs by ulterior centrifugations [109]. Cocktails of proteins were attached to the SeNP surface to act as potential natural stabilizers that prevent the formation of precipitates at the flask’s bottom. Moreover, these SeNPs exhibited nematicidal activity against the nonpathogenic nematode *Steinernema feltiae* and biological activity against *E. coli* and *S. cerevisiae*, for bacterial and yeast infections, respectively. Wadhwani et al. detailed the SeNP synthesis by challenging the cell suspension and total cell proteins (TCP) of *Acinetobacter* sp. SW30 with sodium selenate [58]. This cell suspension formed spherical SeNPs of 78 nm in diameter after 6 h incubation and transformed into rod-like structures after 48 h. These selenium structures were observed at different pH values ranging from 6 to 10 and two precursor concentrations (1.5 and 3.0 mM) (Figure 2). On the other hand, polygonal-shaped SeNPs of 79 nm in size were obtained in the supernatant at 4 mg mL^−1^ of TCP.

Moreover, Fernández-Llamosas et al. reported that the anaerobic beta-proteobacteria *Azoarcus* sp. CIB is tolerant to selenite oxyanions and acts as a good biocatalyst synthesizing electron-dense SeNPs in its stationary growth phase [121]. This study proposed the existence of an energy-dependent selenite exporter to minimize the intracellular accumulation of the as-produced SeNPs by transporting them out of the cell. Tugarova et al. suggested a general mechanism of SeNP biosynthesis by *Aspergillus brasilense* [144]. The process involves the transport of Se ions to the cell interior where they are reduced into elemental Se(0) nuclei; these nuclei are then released to the supernatant where the extracellular biosynthesis of SeNPs occurs. The synergistic inhibition effect of these SeNPs in combination with six antibiotics was tested against pathogenic bacteria. Furthermore, the rhizobacterium *A. brasilense* appears to biotransform selenite to mixed selenium-sulfur NPs with a sulfate concentration of 800 mg L^−1^; this mechanism is suitable for bioremediation, agriculture, nanobiotechnology, and medical applications [125].

Figueroa et al. reported the in vivo and in vitro synthesis of Se and Te nanostructures using *Acinetobacter schindleri* and *Staphylococcus sciuri* from a total of 47 bacterial strains [115]. Triangular, spherical, and rod-like Se nanostructures were also efficiently fabricated in vitro using *E. cloacae* glutathione reductase (GorA) in both crude extracts and purified protein. Similar studies investigated biomolecules involved in mediating the reduction of selenium oxyanions to elemental selenium or SeNPs, such as glutathione (GSH) [140,156,161], glutathione reductase [162], proteins [75,163], thioredoxin reductase [162,164], SerABC reductase [165], fumarate reductase [140,141], NADH-dependent enzymes [166], NADH flavin oxidoreductase [84,166], membrane-bound SrdBCA amino acid sequence [167], DMSO reductase family of molybdoproteins [168], sulfite reductase [169], hydrogenase I [170], nitrite reductase [171], chromate selenite reductase flavoenzyme (CsrF) [75], and other enzymes and biosurfactants [172,173].

In addition, some biomolecules have been found to act as reducing, capping, and/or stabilizing agents and play a fundamental role in altering the features of SeNPs and controlling their size distribution [56,145]. For instance, Ruiz Fresneda et al. indicated that extracellular flagella-like proteins can biotransform the amorphous Se(0) nanospheres to crystalline and polycrystalline one dimensional (1D) trigonal Se(0) nanostructures with distinct shapes, such as nanowires and polygons [74]. Moreover, Wang et al. used *Bacillus subtilis* to obtain semiconducting spherical monoclinic SeNPs that could be transformed into 1-D trigonal nanowires with an actinomorphic nature [101]. This process might involve an oriented attachment mechanism based on the Ostwald ripening mechanism. Moreover, proteins present in the solution are thought to provide long-term stability to the SeNPs and prevent their agglomeration. Another study using *Burkholderia fungorum* strain DBT1 determined aerobic selenite reduction can be attributed to cytoplasmic enzymatic activation mediated by electron donors [122]. The same study suggested that an organic layer surrounding the SeNPs, composed of extracellular matrix (ECM) that includes carbohydrates, proteins, and humic-like substances, stabilizes the particles by modifying their zeta potential.

Previous studies also highlighted the importance of the protein fraction released by microorganisms to externally coat nanoparticles to increase electrostatic repulsions and, consequently, increase their colloidal stability [174,175,176]. This characteristic is essential to maintain the long-term stability, avoid the aggregation and prevent the transformation of colloidal SeNPs into the black amorphous Se form [56]. This is evidenced in high negative z-potential values that are indicative of particle repulsion. For example, carbonyl groups of amino acid residues [142] and SH groups of L-cysteine [177] can strongly bind to metal NPs and form a biomolecular, stabilizing, and protecting cap.

### 5.2. Using Fungi

**Figure 3:**
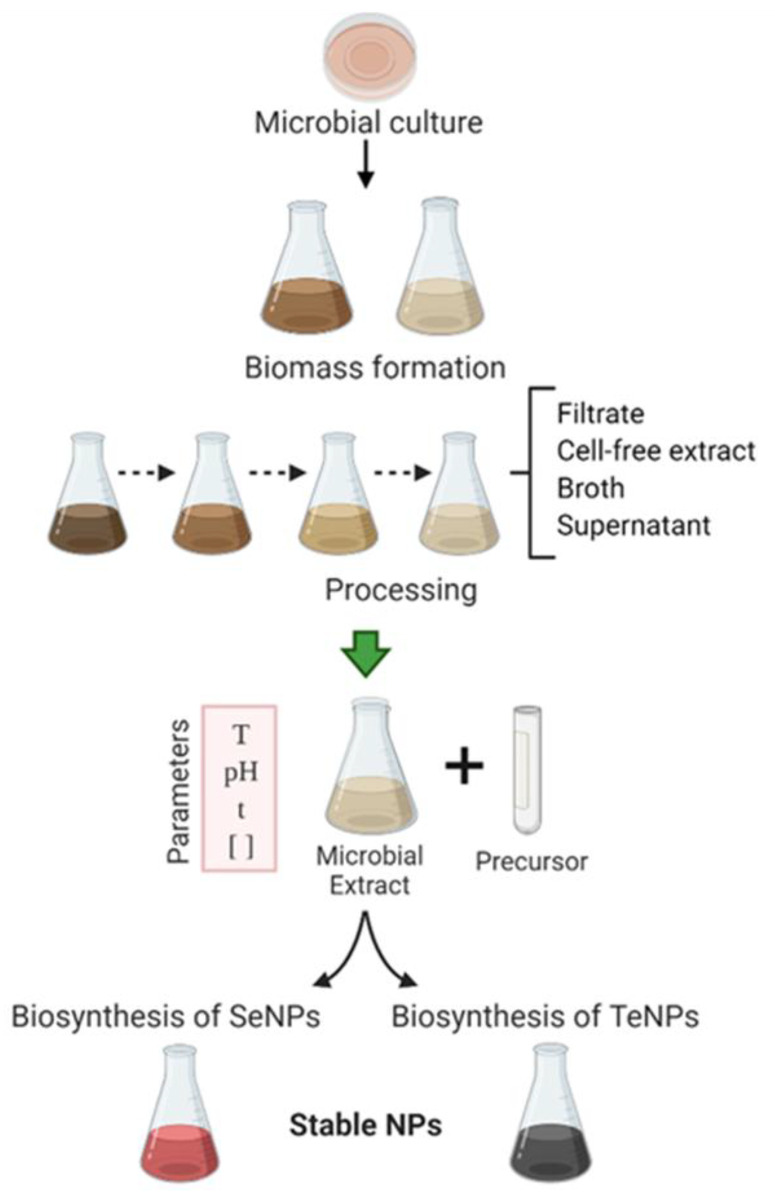
Schematic diagram detailing the microbially assisted procedure of metalloid nanoparticles.

The mycogenic biosynthesis of inorganic NPs has been extensively investigated due to the advantages of fungi over bacteria and actinomycetes [178,179]. Fungi are easy to culture and manipulate, and can grow in highly concentrated media with heavy metal cations. They can also survive and reproduce in high selenium concentrations. The main advantages of NP mycosynthesis are easy scaling-up, low-cost downstream processing and easy manipulation, low-cost and viability of the fungal biomass [180]. Furthermore, fungi release reductive proteins and enzymes into the extracellular medium; these biomolecules reduce Se ions into harmless, precipitating SeNPs [181]. The general process of microbially assisted synthesis of SeNPs and TeNPs is shown in Figure 3.

Numerous fungal species reduce selenite/selenate to intra- or extracellular SeNPs (Table 2). Under extracellular conditions, Diko et al. reported the synthesis of spherical and pseudospherical SeNPs using the supernatant of *Trichoderma* sp. WL-Go in culture broth [182]. Liang et al. used four fungal species: *Aureobasidium pullulans*, *Mortierella humilis*, *Trichoderma harzianum*, and *Phoma glomerata*, to produce SeNPs and TeNPs and provide nucleation sites with extracellular protein and polymeric substances [183]. Mosallam et al. combined γ-rays and the supernatant of *A. oryzae* to produce SeNPs and found a strong correlation between the antioxidant capacity and both the phenolic content and SeNP yield [184]. Moreover, the biomimetic mycosynthesis of SeNPs with simple preparation protocols from, for instance, *Alternaria alternata* yields uniform and stable SeNPs [180].

The medicinal basidiomycete *Lentinus edodes* F-249 can transform selenium within organic and inorganic compounds into spherical SeNPs of ~180 nm [193]. *Dictyophora indusiata* is a saprophytic fungus able to form a hybrid Se nanostructure by exploiting its novel polysaccharide (DP1) [189]. The DP1-functionalized SeNPs proved to have an antiproliferative effect against HepG2 cancer cells via death receptor- and mitochondria-mediated apoptotic mechanisms.

Some studies have also depicted both the intracellular and extracellular synthesis of SeNPs using fungi [191,196]. For example, three fractions of the fungus *Trichoderma atroviride*, namely the culture filtrate (CF), cell lysate (CL), and cell wall debris (CW), produced bioactive SeNPs that were able to form aggregate fungal spores, thus avoiding the adhesion of the pathogen *Phytophthora infestans* to the host cell and blocking its infection of tomato plants [181]. A similar mechanism has been reported for *Lentinula edodes* [193], *Mariannaea* sp. [196], *Fusarium* sp., and *T. reesei* [198]. Other researchers exploited intra- and extracellular extracts of the xylotrophic basidiomycetes *Pleurotus ostreatus*, *L. edodes*, *Ganoderma lucidum*, and *Grifola frondosa* to produce SeNPs of various sizes and shapes [192]. Along with basidiomycetes, other fungal groups, such as *Ascomycota* and *Zygomycota*, can also produce nanoparticles, but these mushrooms are known to be allergenic and/or pathogenic to animals and plants [199,200,201]. Therefore, nontoxic, edible, and cultivated basidiomycetes are a better alternative for biotechnological applications including nanotechnology as the NP synthesis can occur in their mycelia and culture media [192]. Under both extra- and intracellular conditions, the toxicity effects and the removal mechanisms vary according to the fungal species and Se precursors. Rosenfeld et al. demonstrated that six fungal species (*P. sporulosum*, *A. strictum*, *A. alternata*, *P. cucumerina*, *Pyrenochaeta* sp., and *Stagonospora* sp.) constitute an excellent detoxification biosystem that tolerates high Se concentrations and reduces selenite/selenate to Se(0) [191].

El-Sayyad et al. fabricated SeNPs by employing two different eco-friendly green synthetic methodologies: either using *Penicillium chrysogenum* filtrate or combining *P. chrysogenum* filtrate with gentamicin drug (CN) as the stabilizing agent after application of γ-irradiation [186]. The second process resulted in the highest synthesis yield and enhanced antipathogenic and antibiofilm potential. It is also easy to produce Se-based nanocomposites. For instance, Jin et al. prepared SeNPs embedded and homogeneously dispersed in black fungus-extracted BFP nanotubes (triple helix β-(1,3)-D-glucan) that possess hydrophilic hydroxyl groups. These nanocomposites showed interesting cytotoxic and antitumor properties [188].

### 5.3. Using Yeast

Yeast is a relevant model system to investigate the metabolic detoxification pathways of selenite/selenate and their conversion to selenomethionine [202,203]. Thus, Se-rich yeasts are used as a food supplement because they accumulate up to 3000 ppm of selenium [203] and can be used as a cancer treatment at elevated doses (>200 μg Se per day) [204]. However, further analyses are needed to identify and quantify the chemical forms of selenium should these Se-rich yeasts be commercialized. For example, Jiménez-Lamana et al. used single particle inductively coupled plasma mass spectrometry (SP-ICPMS) to detect, characterize, and quantify putative nanoparticles in Se-rich yeasts [205]. Bartosiak et al. calculated the accurate yield of SeNP synthesis mediated by *Saccharomyces boulardii* using continuous photochemical vapor generation (PCVG) coupled with microwave-induced plasma optical emission spectrometry (MIP-OES) and UV-Vis spectrophotometry (PCVG-MIP-OES) [206]. This efficient method enabled the selective identification and quantification of both the unreacted Se(IV) and the final water-soluble SeNPs without the need to separate them. Lian et al. synthesized spherical and quasi-spherical SeNPs of 70–90 nm in size utilizing the yeast cell-free extract of *Magnusiomyces ingens* LH-F1; some surface proteins played a significant role during the synthesis, acting as reducing or capping agents [207]. Nevertheless, the mechanisms of SeNP formation are not fully understood.

*S. cerevisiae* primarily reduces selenium ions through metabolism [208,209]. Owing to its high selenium tolerance, *S. cerevisiae* constitutes a promising and cost-effective alternative for the removal of selenium ions from aqueous solutions [210]. Additionally, it is postulated that SeNPs are expelled from *S. cerevisiae* cells by vesicle-like structures under microaerophilic conditions followed by the ulterior capping of these NPs with residual organic components from the vesicle-like structures [211]. As the SeNPs are stabilized by the natural organic molecules of yeast cultures, there is no need for additional stabilizing agents [206].

The reduction of selenite/selenate to elemental selenium in yeasts forms SeNPs either extra- or intracellularly. In intracellular routes, a genetically engineered, metal-resistant *Pichia pastoris* clone carrying Cyb5R gene has been found to be a safe bioreactor to produce homogeneous and stable selenium and silver NPs. This yeast used a versatile and simple mechanism of biosorption and biotransformation of metals with less toxic waste than physicochemical synthesis [50]. On the other hand, the extracellular processes have the advantage of easy biogenic NP recovery over their intracellular counterparts [211]. According to Rassouli, the general procedure for the extraction and purification of yeast-produced SeNPs consists of (i) applying some enzymatic, chemical, or mechanic method to destroy the cell wall; (ii) collecting the biomass by centrifugation at 8000 rpm for 10 min; (iii) crushing the cells using liquid nitrogen and ultrasounds; (iv) incubating the broken cells with added buffer at 60 °C for 10 min; (v) mixing the pellet containing the cell fragments and NPs with octanol and distilled water to give rise to two phases of which (vi) the SeNP-containing top phase is recovered and further washed with ethanol and chloroform [48].

## 6. Microbial Synthesis of Tellurium Nanoparticles

**Figure 4:**
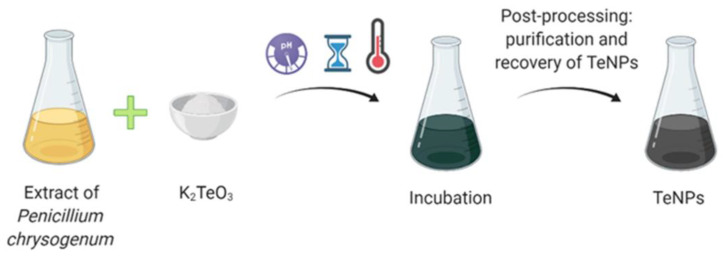
Tellurium nanoparticles (TeNPs) synthesis using microorganisms.

Tellurium is highly toxic to living beings and is not essential in biological metabolism. This may explain why TeNP biosynthesis using microbes is more limited when compared to SeNP [212]. Few articles have been published that detail the biosynthesis of TeNPs using microorganisms (Figure 4) [51,97,213,214,215,216,217,218,219]. Generally, K_2_TeO_3_ or Na_2_TeO_3_ precursors are used to produce TeNPs since they are least toxic when compared to other precursors [97,212,220,221,222]. Tellurium has different oxidation states: telluride (Te^2−^), tellurite (TeO_3_^2−^), tellurate (TeO_4_^2−^). In general, the agglomeration of Te(0) is associated with the respiration of the microorganisms, such as yeast (*S. cerevisiae*), where the fermentation increases the production [223]. On the other hand, a decrease in NP production is observed in bacteria when the oxygen is limited [213].

**Figure 5:**
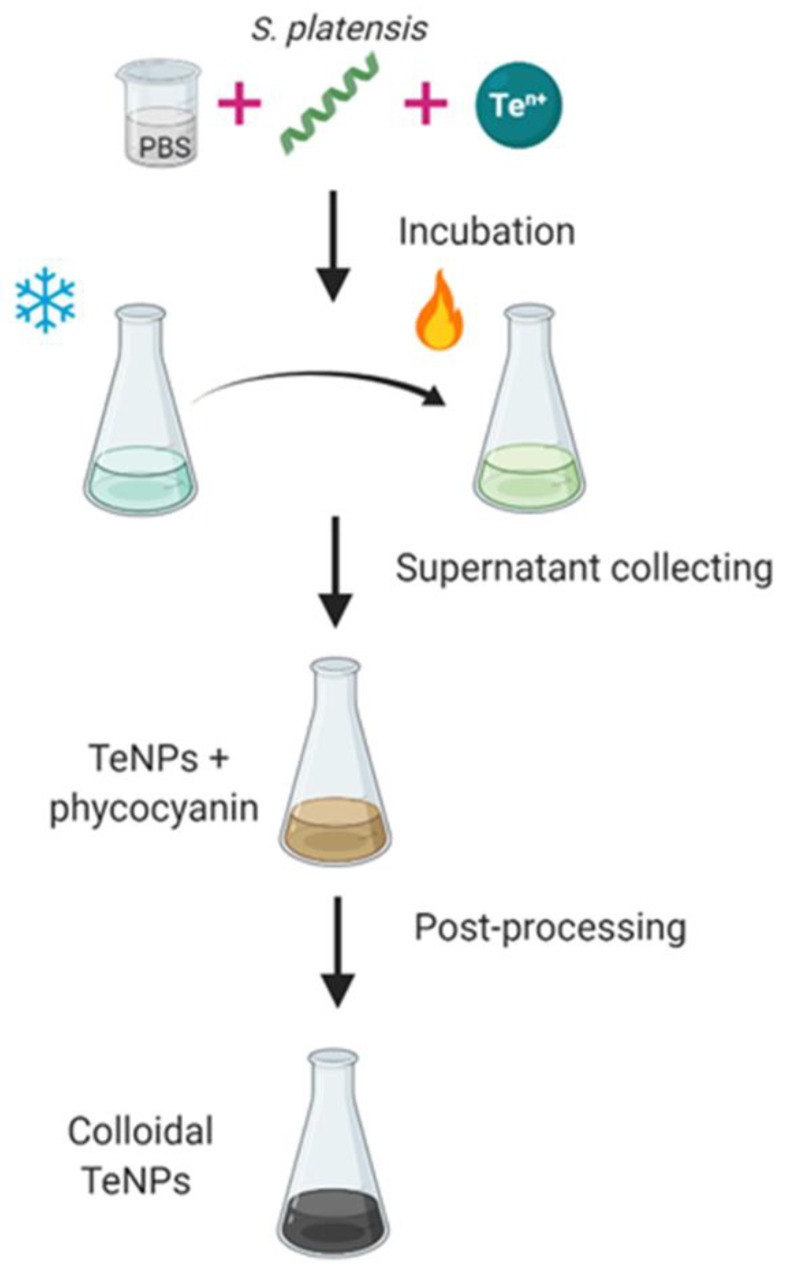
Purification of tellurium-containing phycocyanin (Te-PC) and allophycocyanin (Te-APC) from Te-enriched *S. platensis* using a chromatographic method.

Considering that tellurium is in the same group as selenium, Yang et al. studied the antioxidant activity of TeNPs recovered from tellurium-enriched *Spirulina platensis* cultures where tellurium interacts with two phycobiliproteins, the phycocyanin (Te-PC) and allophycocyanin (Te-APC) (Figure 5) [224].

From a mechanistic point-of-view, a correlation has been established between the growth, size, and shape of TeNPs and the proteins and enzymes present in the media, in addition to other small molecules, such as pyruvate, lactate, and NADH [51,213,225]. Furthermore, the formation of elemental tellurium can be inhibited by other molecules, such as nitrate, nitrite, and fumarate [226]. Since the conditions affecting the TeNP formation can vary as a function of the used organism, there are also great variations in microbe growth time (1–9 days), precursor concentrations (12–600 mg L^−1^), and reaction time (1–8 days).

## 7. Plant-Mediated Synthesis of Metalloid Nanoparticles

Phytonanotechnology is of special interest for synthesizing SeNPs since it is a simple, eco-friendly, high-throughput, and inexpensive route [227,228,229]. The biofabrication of NPs via plants involves proteins, amino acids, organic acids, vitamins, as well as secondary metabolites that act as reducers and stabilizers, such as polysaccharides, alkaloids, flavonoids, phenols, saponins, quinine, steroids, and glycosides [230,231]. Plant-mediated NP synthesis may be carried out through two ways. Via the in vivo route, the NP morphology and size depend strongly on the biosynthesis location, e.g., roots, leaves, fruits, peels, buds, etc., and the implicated metabolites [27]. A chelation-mediated detoxification faculty may explain the mechanism of NP synthesis [232]. The enzymatic antioxidant system is also activated to provide a reactive oxygen species (ROS) balance [233]. Generally, inorganic Se salts (selenite and selenate) taken up by plants are biotransformed into organic Se forms, such as SeCys2, SeMet, and MeSeCys bounded with proteins [234,235]. Hu et al. demonstrated the bioavailability of SeNPs in roots and shoots where they could be biotransformed into organic Se compounds, selenite and selenate to generate Se-biofortified plants [236]. However, the in vitro synthesis using plant extracts is better since it eliminates the lengthy process of cultivation, but still allows for screening the experimental parameters, such as the biomass choice, extraction process and amount, the pH, and temperature [237].

### 7.1. Plant-Based Synthesis of Selenium Nanoparticles

Several papers have reported the plant-derived biosynthesis of SeNPs with varying sizes and morphologies (Table 3). For instance, *Hibiscus sabdariffa* fabricated spherical, triangular, and hexagonal SeNPs with a size of 20–50 nm [238] whereas *Azadirachta indica* has been used as a rapid and efficient biosystem to produce crystalline and spherical SeNPs with a smooth surface [239]. *Withania somnifera* was the best adaptogen herb with active withanolide and flavonoids, used as a bioreductant system to fabricate SeNPs of 40–90 nm [240]. Although plants offer the most suitable green synthesis protocols, the mode of action of plant-produced SeNPs against bacteria remains unknown; it is suggested that the nanoparticles interact with the peptidoglycan layer and break up the bacterial cell wall [227]. Besides, SeNPs are able to induce apoptosis or programmed cell death [174]. Anu et al. reported spherical SeNPs produced by a cheap aqueous extract of garlic cloves, *Allium sativum*, that acted as both the reducing and capping agent [241]. These biogenic SeNPs showed lower cytotoxicity against the Vero cell line than those chemically synthesized. The same group took advantage of the medicinal properties of *Cassia auriculata* to synthesize functional SeNPs that displayed interesting anticancer and antiproliferative characteristics [241]. Similar studies have reported the use of *Vitis vinifera* [32], broccoli extract [108], and *Capsicum annum* [242] to fabricate Se nanorods and nanoballs. Importantly, Ramamurthy et al. presented a combination of SeNPs, made using fenugreek seed extract, and doxorubicin to form a chemoprotective agent against cancer [243]; Vennila et al. studied the antibacterial, anticancer, and anti-inflammatory activity of SeNPs biofabricated by *Spermacoce hispida* and functionalized with apigenin, quinoline, quinazoline, and synaptogenin B [244]; Kokila et al. reported on Se-NPs using the leaves of *Diospyros montana* as a biocidal agent against both Gram+ *S. aureus* and Gram– *E. coli* and the fungus *A. niger* [245].

The application of SeNPs in toxicological studies is relevant due to their association with DNA cytosine methylation, chromatin structure, and transcription processes. It is advantageous for the manipulation and study of cellular division, tissue differentiation, metabolism, and transcription programs [246]. Cui et al. (2018) reported on the production of monodispersed and stable SeNPs from hawthorn fruit extract (HE-SeNPs) whose antitumor activity was evidenced by the apoptosis induced in HepG2 cells through the overproduction of intracellular ROS and mitochondrial membrane potential (MMP) loss or disruption [247]. Additionally, HE-SeNPs induced the upregulation of caspase-9 and downregulation of Bcl-2. Fardsadegh et al. detailed the hydrothermal synthesis of SeNPs using *Aloe vera* leaf extract and determined a prediction model and optimal conditions using response surface methodology (RSM) [249].

### 7.2. Plant-Based Synthesis of Tellurium Nanoparticles

Tellurium is not essential for plant metabolism besides being toxic in most cases [256]. Despite this, it has been documented that some plants have the ability to metabolize Te and transform it into telluroamino acids [257] and organotellurium [258]. *A. sativum*, commonly known as garlic, can assimilate chalcogens to give rise to Te-methyltellurocysteine (MeTeCys) and S-methyltellurosulfide metabolites [256]. The TeNP size is found to be 40–55 nm. The majority of these metabolites were found highly concentrated at the tips of their gloves and in the initial part of the roots. In some cases, TeNPs produced by plants may appear as spheres, rod-shaped, and plates [259].

## 8. Biosynthesis of Bimetallic Se-Te Alloy Nanoparticles

**Figure 6:**
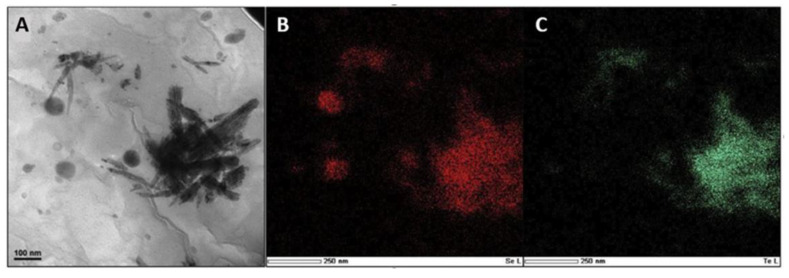
(**A**) TEM image of the hyphae of *Phanerochaete chrysosporium* that depicts Se-Te alloy NPs. STEM-EDS elemental mapping for Se (**B**) and Te (C) that confirms the alloy character of these Se-Te NPs. Adapted from [65] with permission from Elsevier.

Bimetallic Se-Te alloy NPs possess unique and enhanced properties including optical, semiconductive electroresistance, and magnetoresistance [90,260,261]. A few studies have reported the bacterial synthesis of Se-Te nanostructures by *B. beveridgei* [262] and soil isolates of heterotrophic aerobic bacteria [263]. The simultaneous formation of trigonal-hexagonal Se(0)–Te(0) nanostructures from the bioreduction of Se and Te oxyanions in a lab-scale upflow anaerobic sludge blanket reactor (UASB) was also described [149]. A layer of extracellular polymeric substances (EPS) capped the nanoparticles to immobilize them in the granular sludge. Besides crystalline hexagonal TeNPs, the fungus *Phanerochaete chrysosporium* biofabricated unique Se-Te nanospheres and needle-like nanoparticles of 500–600 nm (Figure 6) [65].

Additionally, Asghari-Paskiabi et al. reported the formation of stable Se-S NPs inside *S. cerevisiae* [209]; Vogel et al. investigated the extracellular synthesis of Se-S NPs by *Azospirillum brasilense* mainly attributable to the high negative surface charge due to the covering organic layer made of proteins and carbohydrates [125].

## 9. Bioapplications of SeNPs and TeNPs

In the field of nanobiotechnology, nanoparticles represent the core of a nano-biomaterial; they can be functionalized with different moieties to reduce the toxicity and improve the effects of the drugs [264,265,266]. Moreover, nanoparticles can be used for various medical, industrial, or biological applications. For instance, in nanomedicine, a wide number of surface structures to functionalize the NP surface have been developed for imaging, sensing, and drug delivery applications [267]; the as-obtained NPs can be used for the detection of pathogens and biomolecules or the hyperthermia treatment of cancer [268].

Nanoscale selenium has attracted the attention of scientists due to its bioavailability and lower toxicity compared to the other forms of selenium [269]. Gao et al. studied the antioxidant properties of SeNPs and demonstrated the reduced risk of selenium toxicity [187]. Moreover, SeNPs can be used as an antioxidant in food additives due to their lower risk of toxicity. Besides their antioxidant activity, SeNPs are also an excellent chemopreventive agent against cancer as well as a potential anticancer drug [270]. Specifically, the efficacy and specificity of using nanoselenium at a concentration as low as 2 μg mL^−1^ against prostate cancer has been reported [174]. Other studies highlighted the antimicrobial properties [114] and antifungal activity [271] of SeNPs.

**Figure 7:**
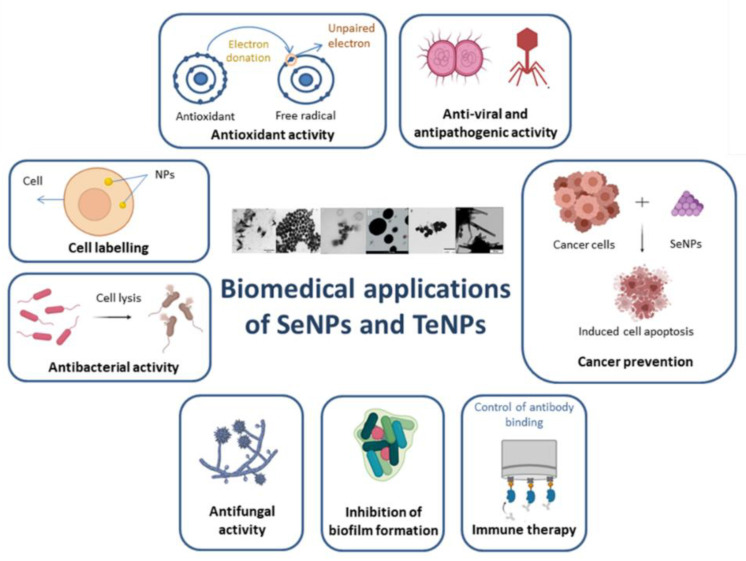
Schematic representation of the various bioapplications of biogenic SeNPs and TeNPs.

The antimicrobial, antioxidant, antifungal, and anticancer properties of TeNPs have been well documented. For instance, Shakibaie et al. described the antioxidant and antimicrobial properties of biologically synthesized tellurium nanorods (TeNRs) [272]. Moreover, another study reveals the antimicrobial and anticancer properties of citrus juice-mediated synthesized TeNPs [62] while the *S. baltica*-synthesized TeNRs exhibit an excellent photocatalytic and anti-biofilm activity to counter potential human pathogens [59]. The next graphic summarizes the main applications of SeNPs and TeNPs (Figure 7).

## 10. Human Cell-Cytotoxicity and Immune Response Induced by SeNPs and TeNPs

According to several studies, various nanoparticles may be cytotoxic and cause harmful effects or even irreversible damage to human cells [264,265]. Therefore, it is necessary to determine how synthesized nanoparticles affect the immune cells [273,274,275]. Selenium nanomaterials have attracted considerable attention as a novel anticancer and chemopreventive agent due to their exceptional biocompatibility and low toxicity [276]. For instance, Cremonini et al. studied the effect of biogenic SeNPs synthesized using *Stenotrophomonas maltophilia* (−) and *B. mycoides* (+) on the viability and function of the antigen-presenting cells, DCs, and cultured fibroblasts (nonimmune cells) [114]. As a result, the as-produced SeNPs did not cause any damage to human cells since there was no stimulation or increase in the release of proinflammatory and immunostimulatory cytokines including IL-12, IL-6, IL-8, and TNF-α. Other studies indicate the SeNPs synthesized by bacteria can induce apoptosis or inhibit both growth and proliferation of cancer cells in culture [276,277,278,279]. SeNPs synthesized by *Acinetobacter* sp. SW30 seem to display a greater anticancer activity when compared to their chemically synthesized counterparts; in fact, they reveal a strong antiproliferative activity against 4T1 cells, MCF-7, NIH/3T3, and HEK293 cell lines [58]. SeNPs synthesized by *B. oryziterrae* also showed potential anticancer activity against H157 lung cancer cell lines [280].

An assay carried out using the SeNPs produced by *Bacillus* sp. MSh-1 against the human fibrosarcoma cell line (HT-1080) demonstrated that the higher the concentration, the higher the cytotoxicity [117]. Moreover, the same study showed the anti-invasive property of HT-1080 cells and the moderate inhibition of MMP-2 expression, a good insight for the treatment and prevention of tumor metastasis. The MTT assay has been used to assess the cell viability, proliferation, and cytotoxicity of breast cancer cells.

One possible explanation for the anticancer activity of SeNPs was reported by Ahmed et al. which encompasses the mobilization of endogenous copper, possibly chromatin-bound copper, and the subsequent prooxidant action [276]. The authors suggested that cancer cells are more subject to electron shuttling between copper ions and selenium nanostructures which release reactive oxygen species (ROS) and thereby kill cancer cells such as Hep-G2 and MCF-7 cell lines. The precise mechanism of anticarcinogenic actions of SeNPs is not totally understood. Since it possesses a high bioactivity and represents the major component of selenoproteins, selenium may increase the carcinogen detoxification, inhibit tumor cell invasion and angiogenesis, enhance immune surveillance, and provide antioxidant protection [281,282,283].

The cytotoxic effects of biosynthesized TeNPs have also been investigated due to their ability to act as an anticancer and antiviral agent [283,284,285]. For instance, Forootanfar et al. demonstrated the lower cytotoxic effect of biogenic TeNRs compared to potassium tellurite on four cell lines of MCF-7, HT1080, HepG2, and A549 [286]. Overall, the toxicity of Te nanostructures depends on the employed synthesis method and their size/morphology [287].

## 11. Conclusions and Perspectives

The present review extensively describes different green methodologies used for the biofabrication of SeNPs and TeNPs. A variety of microorganisms, such as bacteria, fungi and yeast, and plant extracts have become novel, sustainable, risk-free, and cost-effective bionanofactories that reduce selenite/selenate and tellurite/tellurate into their nanosized zero-valent counterparts. To achieve simple, fast, and efficient biological syntheses, these eco-friendly procedures leverage the different organic molecules and metabolites that act as reducing, chelating, and stabilizing agents, such as proteins, EPS, lipids, flavonoids, phenols, and alcohols. The bioreduction and biotransformation of different Se and/or Te species into elemental Se/Te have emerged as an important pursuit in biomedicine, chemistry, nanotechnology, and engineering. Some experimental parameters including the pH, temperature, reaction time, and precursor concentration, along with biosurfactants, play an active role in determining the shape, size, crystallinity, dispersion, and properties of the as-obtained metalloid NPs. This review found that most of the biogenic SeNPs were spherical while their TeNP counterparts were rod-shaped; this constitutes a remarkable outcome in bionanotechnology. However, it is necessary to carry out deeper research on the specifically involved production and transformation mechanisms. Although the toxicity effect of bioresources (i.e., plants) or the nanoparticles synthesized have not been fully explored yet, green production opens up opportunities to manufacture safer nanomaterials and foster better understanding of safety, health, and environment issues.

A myriad of literature shows research at the laboratory scale using living or dead biomass. An important challenge lies in developing large-scale production processes, where larger amounts of templates, surfactants, and other auxiliary substances are required. Then, the use of continuous-flow microreactors and other sources such as waste materials and algae/microalgae may provide significant advantages for industrial level and nanotechnology applications. The development of greener methods that enhance the bioavailability, longevity, and composition-control of NPs could be carried out by computational, synthetic biology and genetic engineering techniques. The employment of natural “nanofactories” is still at an early stage; however, further research would enable the development of straightforward approaches to create potential solutions in nanomedicine, biomedical devices, energy crises, water pollution, and optoelectronics.

## Figures and Tables

**Table 1:** Biosynthesis of SeNPs using bacteria.

Species	Localization	Precursor	Concentration (mM)	Incubation Temperature and Time	Size (nm) *	Color and Shape	Z-Potential (mV)	Sample Quantification	Activity/Application	Ref.
*Staphylococcus carnosus*	Intracellular	Na_2_SeO_3_	1−5	37 °C for 72 h	439–525	Red Spherical	−26.13 and −20.40	Cocktail of proteins derived from *S. carnosus*	Agriculture Future medicine	[109]
*Bacillus mycoides* *Stenotrophomonas maltophilia*	Cell free extract	Na_2_SeO_3_	2	27 °C for 6 h or 24 h	160–171	Spherical	−70 and −80	C: 73–75% O: 10–11% Se: 9–11% P: 3–5% S: 1%	Antibacterial Antibiofilm	[114]
*Acinetobacter schindleri* *Staphylococcus sciuri* *Exiguobacterium acetylicum* *Enterobacter cloacae*	Near the cell membrane	Na_2_SeO_3_	10–50	25 or 37 °C for 24 h	~100	Spherical Transformation to nanowires	N/A	Se: 83.9%	Antibacterial	[115]
*Stenotrophomonas bentonitica*	Intracellular Extracellular	Na_2_SeO_3_	2	28 °C for 48 h	30–400 (~34)	Orange-red Spherical Hexagonal Polygonal Nanowires	N/A	Extracellular flagella-like proteins	Bioremediation, Safety of deep geological repository systems	[74]
*Shewanella* sp.	N/A	Na_2_SeO_3_	0.01–1.0	30 °C for 24 h	1–20	Spherical	N/A	N/A	N/A	[116]
*Bacillus* sp.	Intracellular. Associated to cell debris	SeO_2_	1.26	30 °C for 24 h	80–220	Red Spherical	–16.3	Se: 100%	Anticancer Antibiofilm Antiparasitic Antioxidant	[117,118,119,120]
*Azoarcus* sp.	Extracellular Associated to cell debris	Na_2_SeO_3_	1–8	30 °C for 24 h	123	Orange Spherical	N/A	N/A	Agriculture Bioremediation	[121]
*Acinetobacter* sp.	Intracellular	Na_2_SeO_3_	0.1–4	30 °C for 24 h	~100	Red Spherical Rod shaped polygonal	+10	Proteins Amines Amides	Anticancer	[58]
*Duganella* sp. *Agrobacterium* sp.	Cell surface Extracellular polymeric substances (EPS)Culture medium	Na_2_SeO_3_ Na_2_SeO_4_	4 g L^−1^ 2 g L^−1^	28 ± 2 °C	100–220	Red Spherical	N/A	Proteins	Agriculture	[110]
*Burkholderia fungorum*	Mostly extracellular	Na_2_SeO_3_	0.5–2	27 °C for 96 h	170–200	Red-orange Spherical	From −25 to +20	Proteins	Bioremediation	[122]
*Comamonas testosteroni*	Intracellular: cytoplasm or periplasm	Se(IV) and Se(VI)	5	28 °C for 48 h	100–200	Red fine-grained	N/A	Selenium content 100%	Bioremediation	[123]
*Bacillus subtilis*	Extracellular	Selenite	4	48 °C for 48 h	50–400	Red Spherical monoclinic that can transform to anisotropic 1D trigonal structure (nanowires)	N/A	Proteins Biopolymers	Biosensing	[101]
*Alishewanella* sp.	Intracellular	Na_2_SeO_3_	1	37 °C for 4 h	100–220	Spherical	−28.7	Proteins Lipids Organic substances Inorganic ions	Bioremediation	[75]
*Azospirillum brasilense*	Intracellular Extracellular	Na_2_SeO_3_	10	31 °C for 24 h	50–100	Spherical	−21 to −24	Proteins Polysaccharides Lipids	N/A	[124]
*Azospirillum brasilense*	Extracellular	Na_2_SeO_3_ Na_2_SeO_4_	1–5	30 °C	400	Red Spherical	−18	Proteins Carbohydrates EPS	Bioremediation Biotechnological applications	[125]
*Pseudomonas aeruginosa*	Cell surface	Selenite	0.25–1.0	37 °C for 24–72 h	47–165 (~96)	Red Spherical	251.8	Proteins	Bioremediation	[126]
*Stenotrophomonas maltophilia*	Intracellular Released to the medium	Na_2_SeO_3_	0.5–5.0	27 °C for 24 and 48 h	160–250	Spherical	140	Proteins Carbohydrates Lipids	Bioremediation	[113]
*Bacillus cereus*	Intracellular	Na_2_SeO_3_	0.5–1200	30 °C for 24 h	170	Red Spherical	N/A	N/A	Medicine Veterinary medicine	[127]
*Zooglea ramigera*	Extracellular	Na_2_SeO_3_	3	30 °C for 48 h	30–150	Red Spherical Nanorods (trigonal)	N/A	Enzymes Proteins Bacterial material	N/A	[128]
*Pseudomonas* sp. *Lysinibacillus* *Thauera selenatis*	N/A	Na_2_SeO_3_	200	30 °C for 40 days	N/A	Red Spherical	N/A	Reduced in the presence of nitrate	Denitrification of mine wastewater	[129]
*Escherichia coli*	Intracellular Extracellular	Na_2_SeO_3_	1	N/A	50–100	Spherical	N/A	Quinone-mediated	N/A	[97]
*Acinetobacter* sp.	Intracellular	Na_2_SeO_3_	1	37 °C for 24 h	100 ± 10	Orange Spherical amorphous	N/A	Lignin peroxidase	N/A	[130]
*Enterococcus faecalis*	Extracellular	Na_2_SeO_3_	0.19–2.97	37 and 42 °C for 24 and 48 h	29–195	Red/light red Spherical	N/A	N/A	Antibacterial	[55]
*Streptomyces minutiscleroticus*	Extracellular	Na_2_SeO_3_	1	48–72 h	100–250	Red Spherical	N/A	Proteins	Wound ointment Anticancer drug Coating for medical instruments	[131]
*Streptomyces griseobrunneus*	N/A	N/A	N/A	30 °C	48–136	Red Trigonal	N/A	Proteins Enzymes	Photocatalytic	[132]
*Vibrio natriegens*	Intracellular Associated to cell debris	Na_2_SeO_4_ Na_2_SeO_3_	1	30 °C for 24 h	136 ± 31	Red Spherical	N/A	Proteins	Bioremediation	[133]
*Staphylococcus aureus* Methicillin-resistant *Staphylococcus aureus* (MRSA) *Escherichia coli* *Pseudomonas aeruginosa*	Intracellular Associated to cell debris	Na_2_SeO_3_	2	37 °C for 72 h	90–150	Orange-red	N/A	Lipids Proteins	Antimicrobial	[61]
*Rhodococcus aetherivorans*	Extracellular	Na_2_SeO_3_	0.5–2	40 °C for 40 min then cooled to RT	53–97	Spherical Nanorods	−13 to −32	Organic material	N/A	[99]
*Pseudomonas stutzeri*	Intracellular	Na_2_SeO_3_	2.5	28 °C	100–250	Reddish Spherical	−19.5	Proteins Lipids Other organic substances	N/A	[46]
*Lactobacillus casei*	Intracellular	Na_2_SeO_3_	1.2	37 °C for 24 h	50–80	Red Spherical	N/A	Polysaccharides Proteins	Antioxidant Anticancer	[134]
*Streptomyces enissocaesilis*	Extracellular	SeO_2_	5	30 °C for 72 h	20–211	Brown, orange and deep yellow Spherical	−220	Proteins	Antimicrobial	[135]
*Pseudomonas stutzeri*	N/A	Na_2_SeO_3_	1–3	37 °C for 48 h	75–200	Bright red Spherical	−46.2	Proteins Organic molecules	Antiangiogenic Antiproliferative	[103]
*Streptomyces* sp.	Extracellular	Na_2_SeO_3_	1	28 °C for 72–96 h	20–150	Red Spherical	N/A	Free amines Aromatic rings Cysteine residues Amides	Antibacterial Larvicidal Anthelminthic	[136]
*Lysinibacillus* sp.	Extracellular	Na_2_SeO_3_	1	37 °C for 3 days	130	Red Spherical	−19.1 to −28.8	Proteins Polysaccharides Fatty acids	Antibiofilm Antimicrobial	[137]
*Lactobacillus acidophilus* *L. plantarum* *L. rhamnosus*	Extracellular	Na_2_SeO_3_	4	35° for 48 h	20–80	Red	N/A	Proteins	N/A	[76]
*Idiomarina* sp.	Intracellular	Na_2_SeO_3_	4 and 8	37 °C for 48 h	35 and 150–350	Brick red Spherical/Hexagonal	N/A	N/A	Antineoplastic Anticancer	[138]
*Ralstonia eutropha*	Extracellular	Na_2_SeO_4_	1.5	30 °C for 48 h	40–120	Red Spherical/Nanorods	−7.7	N/A	Antibacterial	[139]
*Pseudomonas stutzeri*	Extracellular Cell surface	Na_2_SeO_4_ Na_2_SeO_3_	5 and 11 mM 4 and 9 mM	34 °C for 7 days	≤200	Red Spherical	N/A	N/A	Bioremediation	[84]
*Enterobacter cloacae*	Intracellular Extracellular	Na_2_SeO_3_	0.5–15	37 °C for 8 h	100–300	Red Rod-shaped	N/A	Organic material	N/A	[140]
*Bacillus cereus*	Intracellular Extracellular	Na_2_SeO_3_	0.5–10	37 °C for 48 h	150–200	Spherical	−46.86	Proteins	N/A	[56]
*Stenotrophomonas maltophilia* *Ochrobactrum* sp.	N/A	Na_2_SeO_3_	0.5	27 °C for 24 and 48 h	357	Spherical	N/A	Organic compounds	Antimicrobial Antibiofilm	[71]
*Shewanella oneidensis*	Cell surface Extracellular	Selenite	0.5	30 °C for 6–48 h	20	Red Spherical	N/A	EPS	N/A	[141]
*Synechococcus leopoliensis*	Intracellular Extracellular	Na_2_SeO_3_	5	35 °C	254 ± 52 200 ± 37	Red-brown Fused spheres Elongated rods	N/A	N/A	N/A	[142]
*Comamonas testosteroni*	Extracellular	Na_2_SeO_3_	0.2–50	28 °C for 24 h	100–200	Red Round Rod-shaped	N/A	Proteins	Bioremediation	[143]
*Azospirillum brasilense*	Extracellular	Na_2_SeO_3_	10–50	31–32 °C for 24 h	25–80	Red-orange Spherical	−21 to −24	N/A	N/A	[144]
*Bacillus cereus*	Cell surface	Na_2_SeO_3_	0.25–1.0	37 °C for 24–72 h	50–150 (~93)	Red Rod-shaped	−31.1 ± 4.9	N/A	Bioremediation	[145]
*Bacillus* sp.	Extracellular	SeO_2_	6.4	33 °C for 72 h	31–335 (~126)	Red-orange Spherical	N/A	Alcohols Phenols Amides Amines Amino acids	Antioxidant	[83]

* An inorganic particle is considered as a nanomaterial if one of its dimensions ranges between 1 and 100 nm.

**Table 2:** Biosynthesis of SeNPs by fungi.

Species	Location	Size (nm)	Shape	Activity/Application	Ref.
*Trichoderma* sp.	Extracellular	20–220	Spherical Pseudospherical	N/A	[182]
*Pleurotus ostreatus*	Aqueous extract	7–28	Spherical	Antioxidant Antimicrobial Anticancer	[185]
*Penicillium chrysogenum*	Cell-free supernatant	48–50	Spherical	Antimicrobial Antibiofilm	[186]
*Phanerochaete chrysosporium*	Intracellular Extracellular	50–600	Spherical	Bioremediation	[65]
*Polyporus umbellatus*	N/A	212 ± 23 82 ± 1	Spherical	Anticancer Antiproliferative	[187]
*Auricularia auricula-judae*	Embedded in triple helix β-(1,3)-D-glucan	60	Hollow nanotubes	Acute myeloid leukemia (AML) therapy	[188]
*Trichoderma atroviride*	Culture filtrate (CF) Cell lysate (CL) Cell wall debris (CW)	60–123	Spherical	Production of crop plants (tomatoes) Management of plant diseases	[181]
*Aureobasidium pullulans* *Mortierella humilis* *Trichoderma harzianum* *Phoma glomerata*	Extracellular	48–78	Spindle-shaped	Bioremediation	[51]
*Dictyophora indusiata*	Intracellular	89	Spherical	Anticancer	[189]
*Catathelasma ventricosum*	N/A	50	Spherical	Antidiabetic	[190]
*Aspergillus oryzae*	N/A	55	Spherical	Antimicrobial	[184]
*Pyrenochaeta* sp. *Acremonium strictum* *Plectosphaerella cucumerina* *Stagonospora* sp. *Alternaria alternata* *Paraconiothyrium sporulosum*	Fungal hyphae Intracellular Extracellular	50–300	Spherical	N/A	[191]
*Alternaria alternata*	Extracellular	30–150	Spherical	N/A	[180]
*Pleurotus ostreatus* *Lentinus edodes* *Ganoderma lucidum* *Grifola frondosa*	Intracellular Extracellular	50–150	Spherical	N/A	[192]
*Lentinula edodes*	Intracellular (fungal hyphae)	180 ± 17	N/A	N/A	[193]
*Pleurotus ostreatus* *Ganoderma lucidum* *Grifola frondosa*	Intracellular Cell-free filtrate	20–550	N/A	N/A	[194]
*Cordyceps sinensis*	N/A	80–125	Spherical	Antioxidant	[195]
*Mariannaea* sp.	Intracellular Extracellular	45 213	Spherical	N/A	[196]
*Gliocladium roseum*	Cell-free filtrate	20–80	Spherical	N/A	[197]

**Table 3:** Different species of plants used for the biosynthesis of SeNPs.

Plant Species	Part	Metabolites	Shape	Size (nm)	Activity/Application	Ref.
*Withania somnifera*	Leaves	Flavonoids Phenolics Tannins	Spherical	40–90	Antibacterial Antioxidant Anticancer	[240]
*Psidium guajava*	Leaves	N/A	Spherical	8–20	Antibacterial	[227]
*Allium sativum*	Cloves	N/A	Spherical	40–100	Cytotoxicity	[241]
*Cassia auriculata*	Leaves	N/A	Amorphous	10–20	Anti-leukemia	[237]
*Momordica charantia*	Roots and shoots	Terpenoids Phenolics	Spherical	10–30	Toxicological studies	[246]
Hawthorn fruit	Fruit	N/A	Spherical	113	Antitumor	[247]
*Hibiscus sabdariffa*	Leaves	Phenols Alcohols	Spherical Triangular Hexagonal	20–50	Antioxidant	[238]
*Pelargonium zonale*	Leaves	N/A	Spherical	40–60	Antibacterial Antifungal	[248]
*Aloe vera*	Leaves	Hydroxyls Amides	Spherical	121–3243	Antibacterial Antifungal	[249]
*Emblica officinali*	Fruit	Phenolics Flavonoids Tannins	Spherical	20–60	Antimicrobial	[228]
*Moringa oleifera*	Leaves	Phenolics Flavones	Spherical	23–35	Anticancer	[250]
*Triticum aestivum*	Roots	N/A	Spherical	140 ± 40	Biofertilizer	[236]
Broccoli	N/A	Carotenes Glucosinolates Polyphenols	Spherical	50–150	Antioxidant Anticancer	[108]
*Diospyros montana*	Leaves	Phenolics Flavonoids	Spherical	4–16	Antibacterial Anticancer	[245]
*Ocimum tenuiflorum*	Leaves	Polyphenols	Spherical	15–20	Inhibition of nephrolithiasis	[183]
*Theobroma cacao*	Shell	Polysaccharides Proteins Phenolics	Spherical Trigonal	1–3	N/A	[251]
*Zingiber officinale*	Roots	Flavonoids Terpenoids	Spherical	100–150	Antimicrobial Antioxidant	[252]
*Mucuna pruriens*	Seed	Phytochemicals	Spherical Nanorods	100–120	Antioxidant Anticancer	[102]
*Azadirachta indica*	Leaves	Polyphenols Flavonoids Proteins	Spherical	142–168 221–328	Antibacterial	[239]
*Vitis vinifera*	N/A	Lignin	Spherical	3–18	N/A	[32]
*Clausena dentata*	Leaves	Flavonoids Triterpenoids Polyphenols	Spherical	46–79	Larvicidal	[229]
*Spermacoce hispida*	Leaves	Polyols Saponins	Rod-shaped	120 ± 15	Anti-inflammatory Antibacterial Anticancer	[244]
*Rosa roxburghii*	N/A	Polysaccharide (RTFP-3)	Spherical	105	Antioxidant	[253]
*Lycium barbarum*	Berries	Flavonols (catechins)	Spherical Triangular	83–160	Antioxidant	[254]
Fenugreek	Seeds	Phenol Flavonol	Oval	50–150	Anticancer	[243]
*Allium sativum*	Bulbs	Alcohols Phenols	Spherical	205	Antioxidant Anticancer	[255]
